# decodeRNA— predicting non-coding RNA functions using guilt-by-association

**DOI:** 10.1093/database/bax042

**Published:** 2017-06-11

**Authors:** Steve Lefever, Jasper Anckaert, Pieter-Jan Volders, Manuel Luypaert, Jo Vandesompele, Pieter Mestdagh

**Affiliations:** 1 Center for Medical Genetics Ghent (CMGG), Ghent University, Ghent, Belgium; 2 Cancer Research Institute Ghent (CRIG), Ghent University, Ghent, Belgium; 3 Biogazelle, Zwijnaarde, Belgium

## Abstract

Although the long non-coding RNA (lncRNA) landscape is expanding rapidly, only a small number of lncRNAs have been functionally annotated. Here, we present decodeRNA (http://www.decoderna.org), a database providing functional contexts for both human lncRNAs and microRNAs in 29 cancer and 12 normal tissue types. With state-of-the-art data mining and visualization options, easy access to results and a straightforward user interface, decodeRNA aims to be a powerful tool for researchers in the ncRNA field.

**Database URL:** http://www.decoderna.org

## Introduction

Recent technological advances have led to the notion that over 80% of the human genome is pervasively transcribed, dramatically increasing the complexity of the transcriptome. In addition to miRNAs that have been the focus of intense research over the last decade, a plethora of long non-coding RNAs (lncRNAs) has been discovered. The latest release of LNCipedia reports over 111 000 annotated human lncRNA transcripts and that number is expected to increase with future RNA sequencing efforts on various tissue and cell types (1). However, whether all these RNA molecules are truly non-coding and functional is still under debate. Unlike miRNAs, lncRNAs primarily regulate gene expression at the transcriptional level by binding and (re-)positioning transcription factors or proteins involved in the regulation of chromatin architecture. Although the mechanisms of lncRNA function are starting to emerge, the pathways and processes downstream of lncRNAs largely remain elusive. This is further complicated by the lack of lncRNA target prediction tools. Such tools are available in the miRNA field and have dramatically accelerated our understanding of miRNA function.

Since functional validation has only been performed for a handful of lncRNAs [184 human lncRNAs in lncrnadb v2.0 (2)], several studies have successfully applied the guilt-by-association principle to infer functions of lncRNAs on a genome-wide scale (3–5). This approach is based on a correlation analysis between matching non-coding RNA and protein coding mRNA expression in combination with enrichment strategies to project functional protein coding gene sets onto mRNAs correlated with the ncRNA of interest (3,6). Previously, our lab published an online database containing such inferred functions for miRNAs called the miRNA body map (4). Similar web tools utilizing enrichment strategies to predict miRNA functions have been published following our original publication (7,8). To our knowledge, only two other web tools—lncRNA2function (57 samples from 2 studies) and co-LncRNA (29 012 samples from 241 independent datasets)—apply this method for lncRNAs. Both tools follow a hypergeometric test-based approach—limited to co-expressed genes—to identify lncRNA functionality. Other, non-enrichment-based approaches to characterize lncRNA functionality include e.g. transcription factor binding (TF2LncRNA) (9), lncRNA/protein and lncRNA/DNA binding (longTarget) (10), differential mRNA expression upon lncRNA modulation (lncRNA2Target) (11) or a combination of lncRNA expression, sequence conservation, coding potential and secondary structure formation (lncRNAtor, lncRNA-MFDL) (12,13).

Here, we present decodeRNA, the successor of miRNA body map. decodeRNA contains inferred functions both for miRNAs and lncRNAs in over 29 cancer and 12 normal tissue types (10 489 samples in total) with novel visualization, data analysis and data interpretation features. Compared to other tools, decodeRNA enables users to (i) retrieve ncRNA-pathway associations and miRNA-target associations in individual datasets as well as across all datasets, (ii) compare these associations between datasets and (iii) retrieve the individual genes contributing to the ncRNA-pathway associations. 

## Results

### Database content

Functional lncRNA and miRNA contexts in decodeRNA are based on matching lncRNA, miRNA and mRNA expression data from The Cancer Genome Atlas (TCGA). Processed data are stored in a MySQL database optimized for convenient data retrieval. At the time of data retrieval, level 3 RNA-seq and small RNA-seq data are available for 10 489 samples representing 29 cancer and 12 normal tissue types. LncRNA identifiers are associated with common gene names and LNCipedia IDs, whereas miRNAs are linked to their official miRBase entry. ncRNA functions are inferred using the GSEA method as reported previously (4) with functional gene set collections obtained from the Molecular Signatures Database (14) including Chemical and Genetic Perturbations (CGP), Pathway Interaction Database (PID) and the BioCarta, KEGG and Reactome pathways. Currently, functional contexts for miRNAs and lncRNAs are based on > 500 million datapoints for 3340 ncRNA genes (2320 lncRNAs and 1020 miRNAs). In total, 98.81% of the lncRNAs and all of the miRNAs present in the TCGA (small) RNA-seq datasets have at least one associated gene set, with an average of 60.41 and 58.15 ncRNA/geneset correlations per tissue type for lncRNA and miRNA, respectively.

### Workflow

**Figure 1.**
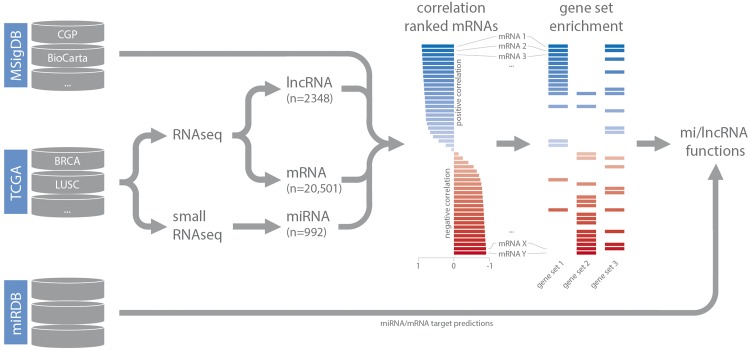
Schematic overview of the analysis workflow and the online repositories used (CGP = chemical and genetic perturbations, BRCA = breast invasive carcinoma, LUSC = lung squamous cell carcinoma).

Small RNA-seq datasets (level 3) were downloaded from TCGA and miRNA read counts were normalized based on total miRNA read counts as reported previously (15). For RNA-seq data, normalized gene expression values (level 3) were downloaded. Expression-correlation matrices (Spearman’s rank) for each dataset were constructed by combining lncRNA and miRNA expression data with matching mRNA expression data. These matrices were used as input for gene set enrichment analysis (in combination with the C2 curated gene set list obtained from MSigDB) (14). For each highly confident ncRNA—gene set association (GSEA FDR < 0.001), gene set FDR, gene set enrichment score, leading-edge mRNAs (up to a maximum of 20 per gene set) and leading-edge mRNA correlation values were stored in separate MySQL tables, based on the type of gene set (CGP, KEGG, BioCarta, Reactome or PID), and coupled to the relevant dataset. As we are working with poly-A enriched RNA-seq data, decodeRNA does not contain information for non-poly-adenylated lncRNAs. For miRNA datasets, miRNA/mRNA target associations, retrieved from miRDB, are stored for inclusion in downstream visualizations (Figure 1) (16).

### Features

The interface allows easy retrieval of various information layers. When using the lncRNA to tissue/gene set workflow to identify potential lncRNA functions, users are guided to the main ncRNA2function page upon the selection of the desired RNA type (lncRNA or miRNA) and submission of a ncRNA identifier. Here, depending on the molecule of interest, two (for lncRNAs) or four (for miRNAs) types of analysis are available. For both lncRNAs and miRNAs, functional contexts (i.e. ncRNA—gene set associations) in all or a selection of the registered datasets can be displayed. The first option will display a form where the functional context query can be customized. The gene set collection and (up to five) datasets of interest can be selected and an FDR cutoff can be set (relevant for further visualization purposes). This enables users to further reduce the number of resulting functional contexts to the most confident ones. Output can be generated in the form of gene set lists with associated FDR-values, a Circos plot displaying the gene sets and corresponding (up to 200) leading-edge genes (defined as the core of a gene set that accounts for the enrichment signal), or a customizable Circos plot enabling the user to select the gene sets to be visualized (17). Edges connecting identical leading-edge genes in different gene sets can be used to appreciate the degree of gene set overlap. All data used to generate the list or Circos plots can be exported as tab-separated text files. Choosing the second option will show the user in what fraction of all available datasets each gene set—correlated with the ncRNA of interest—is present, ranked based on significance, with link-outs to MsigDB for more information regarding the corresponding gene set and the possibility to view FDR-values and enrichment scores for a certain gene set in all of the significant datasets. A second workflow allows users a different entry into the decodeRNA data. Following the selection of a tissue type of interest, lncRNAs associated with at least one gene set (FDR = 0) in that tissue type are retrieved. LncRNAs and their corresponding gene sets are visualized in a list format, ranked according to the number of gene sets associated with each lncRNA. This allows users to identify lncRNAs associated with specific cellular processes or lncRNAs having certain gene set association in common, hinting at potential lncRNA functions.

For miRNAs, two additional analyses, related to the targets of these miRNAs, are available. The first is across all available datasets and displays a list of predicted miRNA targets whose expression is negatively correlated with the miRNA of interest (Spearman rho < −0.5, *P*-value < 0.05), along with the fraction of datasets in which a significant negative expression correlation is observed. The second analysis uses a form to filter miRNA target results according to dataset selection and level of significance. Results are shown in either a list form or by means of a Circos plot, with similar layout as the pathway-option described above. 

### Generating hypotheses using decodeRNA predictions

When focusing on highly significant gene set associations (FDR = 0), the lncRNAs present in decodeRNA have an average of 26.30 associations per dataset. A distribution plot for the number of gene sets associated with each ncRNA across all datasets can be observed in Supplementary Figure S1. For 26 lncRNAs, identical gene set associations can be observed in all available tissue types. Most of these gene sets correspond to global cellular processes such as translation regulation, oxidative phosphorylation, electron transport and ATP synthesis, suggesting these lncRNAs are involved in housekeeping functions, common across tissue types. For three of these lncRNAs (*SNHG5*, *GAS5* and *ZFAS1)*, such functions have indeed been identified (18–20). Alternatively, we can subset the database for more specific gene sets. For instance, when searching for lncRNAs with putative involvement in the *MYC* pathway, 2190 lncRNAs were identified with high correlation (abs(NES)  > = 0.7) to *MYC* associated gene sets, including *SNHG16*, *DANCR*, *USP2-AS1*, *DLEU2*, *SNHG15*, *PVT1*, *CASC11*, *SNHG6* and *VPS9D1-AS1* (21–26). In a similar way, lncRNAs linked to cell proliferation (*n* = 1684, including *TMPO-AS1*, *DEPDC1-AS1*, *HMMR-AS1*, *UHRF1*, *FAM83A-AS1*, *KDM4A-AS1*, *DDX11-AS1* and *DLEU2*) or migration (*n* = 1335, including *DEPDC1-AS1*, *C14orf34*, *FAM83A-AS1* and *HMMR-AS1*) can be identified (27–35). A full list of the lncRNAs with the number of associated migration/proliferation/*MYC* gene sets can be found in Supplementary Table S1. Novel lncRNA—gene set predictions including several ones in Supplementary Table S1 (e.g. *RP11-244M2.1* and *RP11-120D5.1* for proliferation, *LINC00704* and *RP5-1158E12.3* for migration and *RP11-132A1.4*, *RP11-20B24.4* and *FOXD2-AS1* for possible associations with *MYC*) should be validated by means of wet lab experiments. Ideally, these are based on lncRNA perturbation combined with genotypic or phenotypic read-outs tailored to the predicted function.

### Case studies

To evaluate the performance of decodeRNA in making highly confident ncRNA function predictions, case studies for three different ncRNAs—1 miRNA and 2 lncRNAs—were performed.

**Figure 2.**
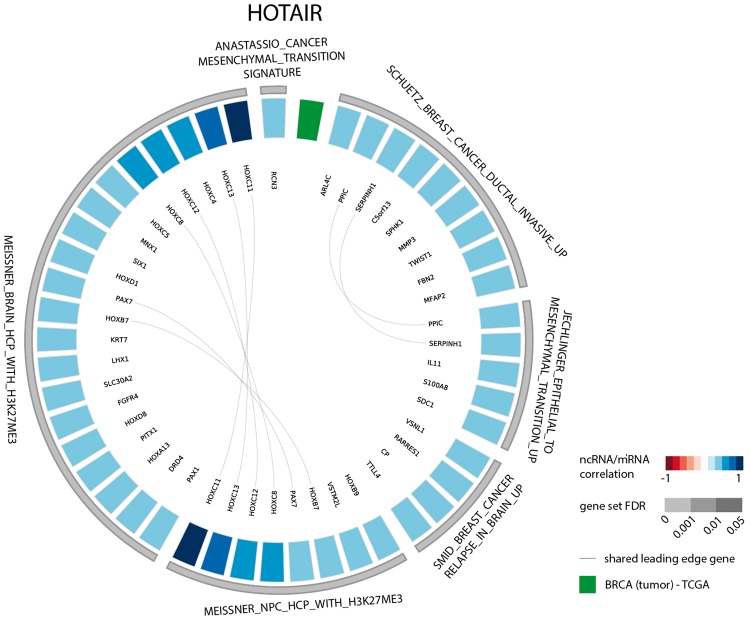
Circos plot representing significant gene sets for the lncRNA *HOTAIR* in breast tumor tissue, focusing on the CGP gene set.

**Figure 3.**
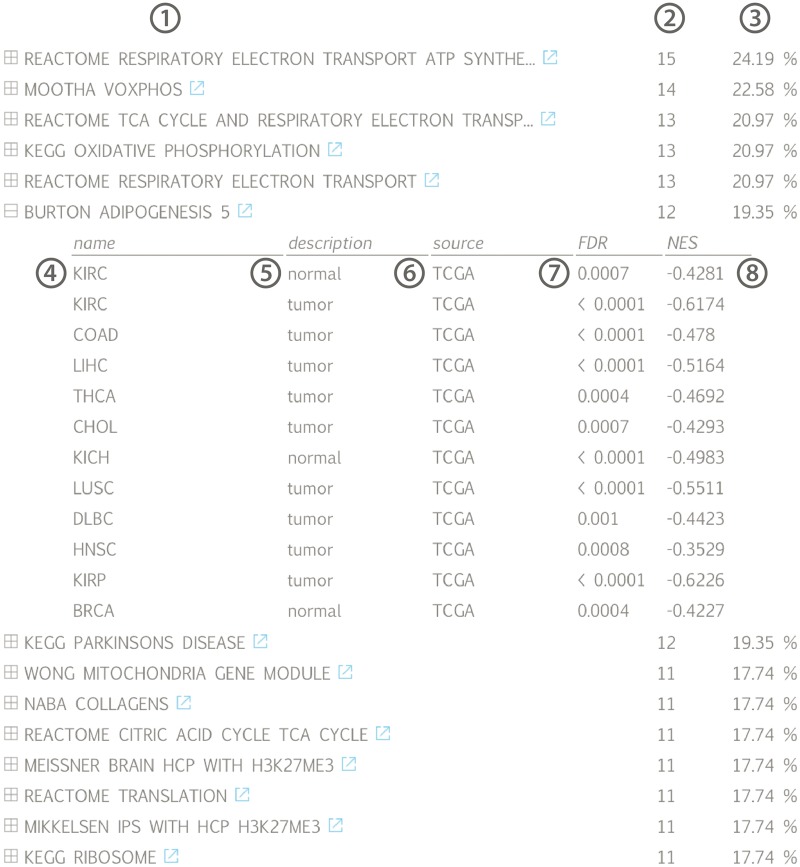
Gene set list across all available datasets for lncRNA *HOTAIR*, ranked according to the fraction of datasets in which a significant association (positive or negative) can be found: (1) gene set name with linkout to MsigDB, (2) number and (3) fraction of datasets in which a correlation with the associated gene set can be found, (4) tissue name, (5) tissue type, (6) data source, (7) gene set false discovery rate and (8) gene set normalized enrichment score; (KIRC = kidney renal clear cell carcinoma, COAD = colon adenocarcinoma, LIHC = liver hepatocellular carcinoma, THCA = thyroid carcinoma, CHOL = cholangiocarcinoma, KICH = kidney chromophobe, LUSC = lung squamous cell carcinoma, DLBC = lymphoid neoplasm diffuse large B-cell lymphoma, HNSC = head and neck squamous cell carcinoma, KIRP = kidney renal papillary cell carcinoma, BRCA = breast invasive carcinoma).

The results of the first evaluation are shown in Figure 2 and display the most significant positively correlated gene sets for the lncRNA *HOTAIR* in breast cancer. Among those are several gene sets related to H3K27 methylation that is induced by *HOTAIR* through recruitment of *PRC2*. In addition, a gene set containing genes down regulated in non-metastasized breast cancer—and thus up regulated in metastasized breast cancer—as well as a gene set related to genes involved in epithelial to mesenchymal transition confirms the experimentally validated association between *HOTAIR* and breast cancer metastasis (36,37). When looking at the list of correlated gene sets for lncRNA *HOTAIR* across all datasets, we again observe various H3K27me3 related gene sets and several gene sets corroborating the recently identified role of *HOTAIR* in adipogenesis (Figure 3) (38).

**Figure 4.**
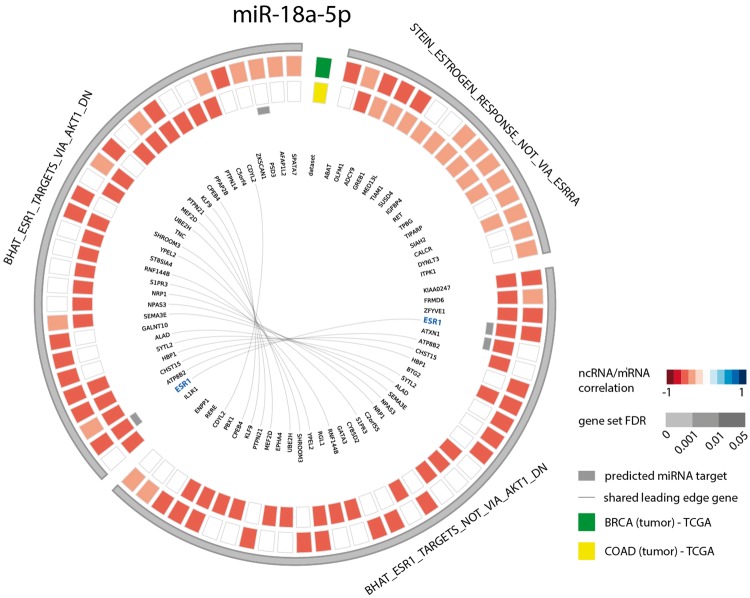
Circos plot representing significant *ESR1* associated gene sets for microRNA hsa-miR-18a-5p in breast and colon cancer (COAD = colon adenocarcinoma, BRCA = breast cancer).

As a second example, we looked at the gene sets correlated with miR-18a-5p in both breast and colon cancer. Since several studies have identified this miRNA to be a regulator of *ESR1* in these cancer types (39–42), we evaluated miR-18a-5p functional contexts in the decodeRNA output. This association was indeed confirmed with three *ESR1* associated gene sets in the resulting gene set list, all of which ranked in the top 25 for both datasets (Figure 4).

**Figure 5.**
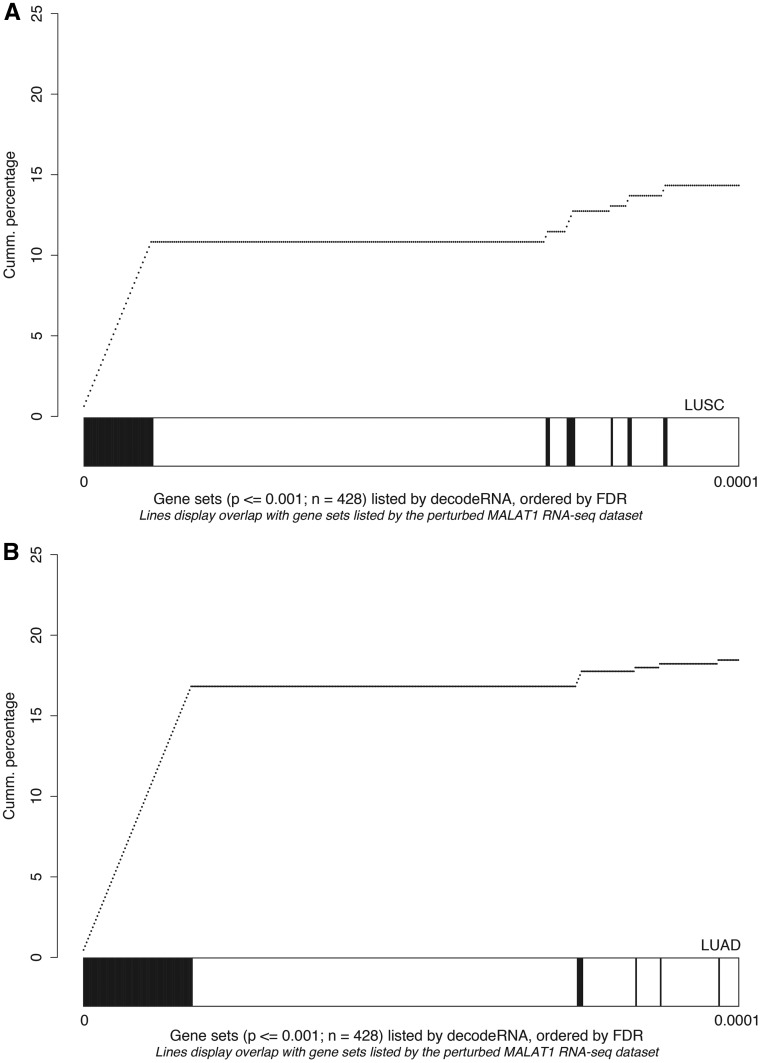
Gene set overlap between the *MALAT1* perturbation experiment in lung cancer and decodeRNA output for *MALAT1* in A) lung squamous cell carcinoma (LUSC,) and B) lung adenocarcinoma (LUAD) datasets. The line-graph displays the cumulative distribution of the gene set overlap in function of the FDR value, while the bars show the position of decodeRNA gene sets in the ranked list of gene sets obtained from the public *MALAT1* perturbation dataset.

As a third and final test to evaluate the decodeRNA functionality, we performed GSEA on a publically available RNA sequencing dataset obtained upon *MALAT1* perturbation in lung cancer (GEO accession number GSE43830) and determined the overlap of the resulting gene sets (upon perturbation) with the ones obtained through decodeRNA for *MALAT1* in lung adenocarcinoma (LUAD) and lung squamous cell carcinoma (LUSC). Both for the LUAD and LUSC datasets, gene sets identified by decodeRNA significantly overlapped with those identified from the MALAT1 perturbation experiment (Fischer exact test, LUAD: *P* < 2.2e-16; LUSC: *P* = 0.003042). Of note, a higher degree of overlap was seen for the most significant decodeRNA gene sets, suggesting that the FDR-values in decodeRNA can be applied to further prioritize gene sets (Figure 5). In addition, the overrepresentation of cell cycle-, cancer-, proliferation- and *B-MYB*-related gene sets corresponds to the validated *MALAT1* functionality reported in the literature (43–45). This, together with the results of the *HOTAIR* and miR-18a-5p case studies, underscores the ability of decodeRNA in making accurate predictions over a broad range of ncRNAs and tissues, helping researchers in providing a functional context for their ncRNA molecules of interest.

## Discussion/conclusion

The current version (1.0) of decodeRNA contains 12 normal and 29 cancer tissue types representing a total of 10 489 samples. This vast amount of data for both miRNAs and lncRNAs, with pre-computed functional context information across a wide variety of tissue types, can guide researchers in setting up wet lab experiments to further elucidate the functions of their ncRNAs of interest. As decodeRNA functional contexts are predictions, experimental validation remains an essential step of the workflow. The aim of a functional context is to provide clues on putative functions and, as such, guide the selection of a relevant functional readout or model system. In future updates, we plan to add additional datasets and expand currently available datasets by reanalysis of (raw) level 1 (small) RNA-seq data from TCGA in order to extend the number ncRNAs. With functional context information for >3300 ncRNAs in over 40 data sets, decodeRNA could be a good starting point for future studies on ncRNA function.

## Supplementary data

Supplementary data are available at *Database* Online.

## Supplementary Material

Supplementary Data
