# Supplementary figures and images for: decodeRNA— predicting non-coding RNA functions using guilt-by-association
Source: Database (Oxford). 2017 Jun 11;2017:bax042. doi: 10.1093/database/bax042 (PMC5502368; doi:10.1093/database/bax042)

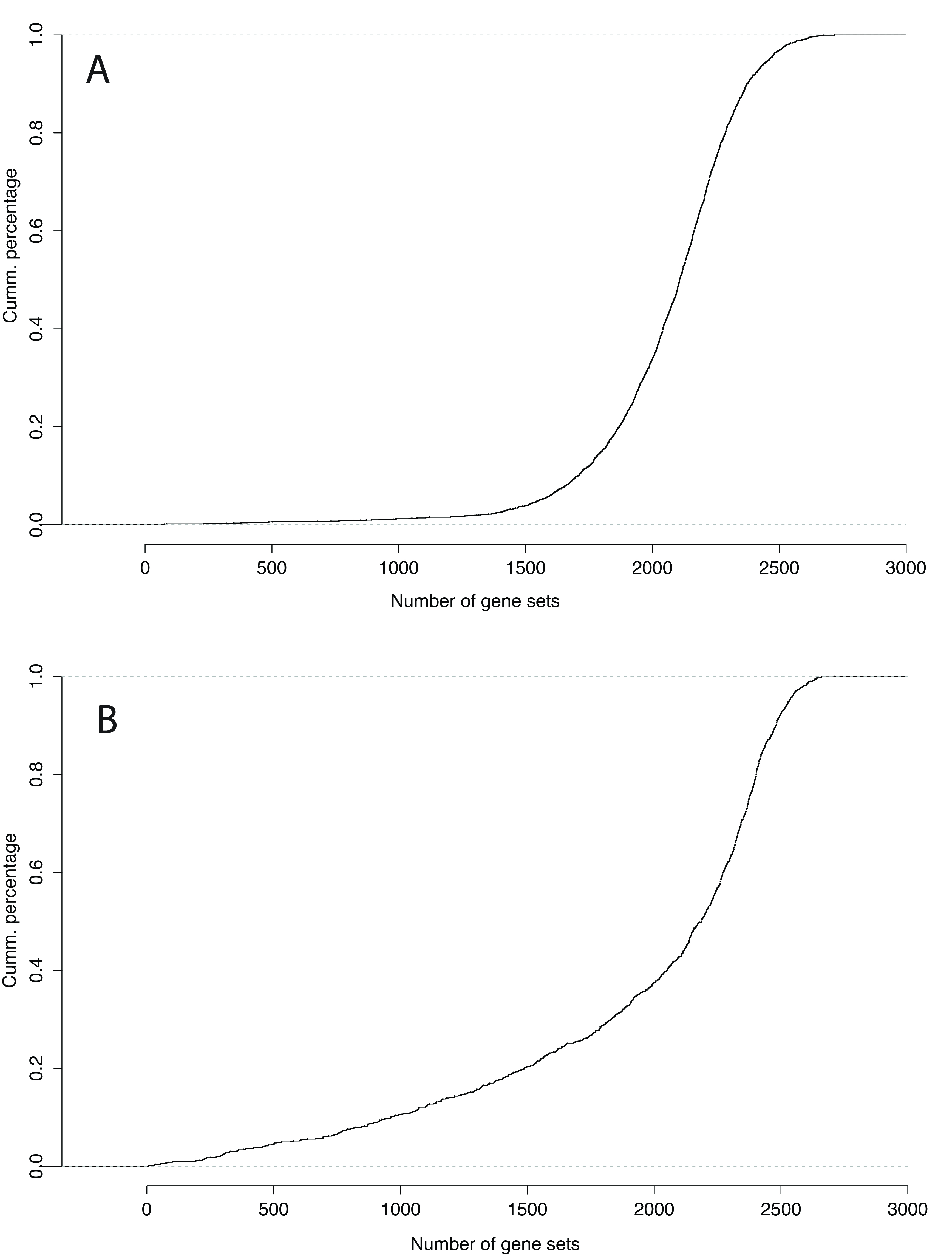

Supplement: Supplementary Data [file bax042_Supp.zip › suppl_figure1.jpg]
